# Interventions for Fontan Pathway Obstruction in Patients Following Total Cavopulmonary Connection

**DOI:** 10.3390/jcm14207447

**Published:** 2025-10-21

**Authors:** Nicole Piber, Christina Ruda, Thibault Schaeffer, Muneaki Matsubara, Jonas Palm, Teresa Lemmen, Paul Philipp Heinisch, Stanimir Georgiev, Alfred Hager, Peter Ewert, Markus Krane, Jürgen Hörer, Masamichi Ono

**Affiliations:** 1 Department of Cardiovascular Surgery, TUM University Hospital German Heart Center, 80636 Munich, Germany; 2 Department of Congenital and Pediatric Heart Surgery, TUM University Hospital German Heart Center, 80636 Munich, Germany; 3 Division of Congenital and Pediatric Heart Surgery, University Hospital of Munich, Ludwig-Maximilians-Universität München, 81377 Munich, Germany; 4 Europäisches Kinderherzzentrum München, 81377 Munich, Germany; 5 Department of Congenital Heart Disease and Pediatric Cardiology, TUM University Hospital German Heart Center, 80636 Munich, Germany; 6 Partner Site Munich Heart Alliance, DZHK (German Center for Cardiovascular Research), 80636 Munich, Germany

**Keywords:** total cavopulmonary connection, Fontan pathway obstruction, pulmonary artery, extracardiac conduit, stent implantation

## Abstract

**Background/Objectives**: Obstruction of the Fontan pathway is a severe morbidity after total cavopulmonary connection (TCPC). This study aimed to evaluate the incidence and location of TCPC pathway obstruction and corresponding interventions. **Methods**: In all patients undergoing TCPC between 1994 and 2023, postoperative interventions for TCPC pathway obstruction were evaluated. Risk factors for TCPC pathway interventions were identified, and the impact of TCPC pathway interventions on late outcomes was analyzed. **Results**: Among 650 patients, 136 (21%) needed catheter/surgical interventions for TCPC pathway obstructions during the median duration of 0.2 (0.03–6.1) years postoperatively. Interventions comprised 128 catheters and 10 surgeries. Catheter intervention included 107 left pulmonary arteries (PA), 8 right PAs, and 27 extracardiac conduits. Surgery included eight conduit revisions, four PA enlargements, and two SVC enlargements. Freedom from interventions at 1, 3, 5, and 10 years was 87.7, 85.3, 83.6, and 78.5%, respectively. Previous Norwood procedure (HR: 2.228, *p* = 0.003), previous ductal stent (HR: 2.574, *p* < 0.001), previous PA interventions (HR: 2.514, *p* < 0.001), and high PA pressure before TCPC (HR: 1.161, *p* = 0.004) were risk factors. Patients requiring interventions had a higher incidence of protein-losing enteropathy (16.0 vs. 2.0%, *p* < 0.001), plastic bronchitis (8.3 vs. 0.8%, *p* < 0.001), and failing Fontan (28.6 vs. 7.6%, *p* < 0.001), compared to those who did not. **Conclusions**: Interventions for Fontan pathway obstruction were needed in 21% of patients. The left-PA stenosis was the main lesion, most cases of which were treated by stent implantation. Norwood procedure, ductal stent, pre-TCPC PA intervention, and high pre-TCPC PA pressure were identified as risks factors.

## 1. Introduction

The Fontan operation, established by Fontan and Baudet in 1971, describes the connection of the systemic venous flow to the pulmonary arteries (PA), predominantly used in patients with tricuspid atresia [1]. Over more than fifty years, surgical treatment of patients with univentricular heart has evolved until today. Nowadays, the total cavopulmonary connection (TCPC) is a well-established and safe treatment that improves the survival of patients with univentricular hearts [2,3,4,5]. Currently, the 30-year survival after TCPC is about 85% [6,7,8]. After the introduction of the staged Fontan strategy and fenestration, more complex patients with worse conditions reached TCPC. Thus, the focus has shifted from short-term survival to long-term durability of the Fontan circulation. An unobstructed Fontan pathway is essential for optimal hemodynamics, as the Fontan circulation relies on low-resistance, “passive” flow to the PA. Therefore, Fontan pathway obstruction is a serious complication characterized by an anatomical or functional narrowing anywhere in the TCPC pathways. The Fontan pathway can become stenotic by several mechanisms: longitudinal stretch, torsion, internal calcification, thrombosis, and neo-intimal growth [9]. Previous studies demonstrated that the obstruction of the Fontan pathway affects approximately 15% of patients after TCPC [10,11]. Compression or narrowing of the left pulmonary artery may result from the presence of ductal tissue extending into the artery due to tissue retraction, external pressure on the artery following complex aortic surgeries (such as the Damus–Kaye–Stansel (DKS) anastomosis or the Norwood procedure), narrowing near the origin of the pulmonary artery caused by a right ventricle-to-pulmonary artery conduit, or distortion of the artery due to deformities from previous palliative banding [11]. Conduits made from Dacron material are associated with a higher risk of obstruction [12]. As a therapeutic option, stent implantation in the Fontan pathway has emerged as a promising strategy, potentially avoiding complex surgical procedures [13,14,15]. However, the occurrence and treatment of Fontan pathway obstruction are not fully understood, especially regarding location and therapy options in a large cohort. In this study, we analyzed patients who underwent TCPC at our institution, focusing on the occurrence of Fontan pathway obstruction and performed treatments. We aimed to assess the correlation of patient factors and intervention on Fontan pathway obstruction, comparing interventional and surgical treatment options to provide guidance for the right decision in future treatment of Fontan pathway obstructions.

## 2. Materials and Methods

### 2.1. Data Availability Statement

The data that support the findings of this study are available from the corresponding author upon reasonable request.

### 2.2. Ethical Statement

The study was approved by the Institutional Review Board of the Technical University of Munich (approval number 2025-232-S-NP on 5 May 2025). Due to the retrospective nature of the study, the requirement for individual patient consent was waived.

### 2.3. Patients and Data Collection

This retrospective study, conducted at a single center, analyzed all patients who underwent total cavopulmonary connection (TCPC) between 1994 and 2023. Individuals who had a conversion from a classic Fontan procedure to TCPC were not included in the analysis. Baseline and follow-up data for patients were obtained from our single ventricle database, which includes outcomes from the later stages of palliative treatment. These include the initial stage I palliation characterized by the Norwood procedure, aorto-pulmonary shunt, pulmonary artery banding, and patent ductus arteriosus (PDA) stenting followed by stage II bidirectional cavopulmonary shunt (BCPS), and TCPC. The follow-up duration for each patient was measured from the date of their TCPC procedure to their most recent clinical evaluation, with follow-up concluded on 31 October 2024. Only surgical and catheter procedures were evaluated as interventions for TCPC pathway obstructions. Other interventions, such as closure of fenestration, creation of fenestration, closure of residual baffle shunt, and closure of collateral vessels, were not included.

### 2.4. Surgical Techniques

TCPC was performed through a median sternotomy on cardiopulmonary bypass with aortic and bicaval cannulation. Lateral tunnel TCPC was performed between 1994 and 2022, according to the technique introduced by de Leval et al. [3], and a polytetrafluoroethylene graft (Gore-Tex) was used for the intra-atrial baffle. Since 1999, extra-cardiac TCPC was performed using a non-ringed Gore-Tex tube graft [4,5,16]. Operations were usually performed in normothermia. Routine modified ultrafiltration was performed after weaning from cardiopulmonary bypass. Fenestration was not routinely performed, only in high-risk constellations, such as single-lung Fontan or reduced ventricular function [17].

### 2.5. Cardiac Catheterization and Intervention Techniques

Cardiac catheterization was routinely performed before TCPC. Hemodynamic measurements of the pulmonary artery pressure (PAP) and the left-atrial pressure (LAP) were performed, and the trans pulmonary gradient was calculated. Other parameters, including the systemic ventricular systolic pressure, the end-diastolic pressure, the mean arterial pressure, and the arterial oxygen saturation, were measured. The PA index was calculated using PA angiography as described by Nakata and colleagues [18]. The right- and left-PA indices were calculated by dividing the cross-sectional area of each PA branch by the body surface area. To evaluate the symmetric PA development, the symmetry index was calculated as described by Glatz and colleagues [19]. For further evaluation of the symmetric PA development, the ratio of the left to the right-PA index was calculated by dividing the left-PA index by the right-PA index. Postoperatively, cardiac catheterization was not routinely performed and was only indicated when patients were symptomatic or a hemodynamic evaluation was needed. All interventional procedures (balloon/stenting) for TCPC pathway obstruction were performed by a senior cardiologist who is highly experienced and specialized in pediatric and congenital interventional cardiology. The stenting procedure was performed in the presence of significant PA/SVC/IVC/conduit stenosis (50% or more reduction) using balloon-expandable stents. Stents were chosen under the consideration of post-dilating them to compensate for the somatic growth in the follow-up [20]. The presence of left-bronchial compression caused by the stents was closely monitored following stent implantation and throughout the follow-up period as part of standard evaluations.

### 2.6. Definition of Failing Fontan, Protein-Losing Enteropathy, and Plastic Bronchitis

In this study, failing Fontan was diagnosed on the basis of clinical symptoms. Based on Kramer et al.’s study, failing Fontan was defined as having at least one of the following criteria: 1. New York Heart Association (NYHA) functional class IV heart failure, 2. persistent NYHA functional class III heart failure for ≥12 months without sustained improvement, 3. more than 2 unscheduled hospital admissions within 12 months due to heart failure symptoms, 4. evaluation or listing for heart transplantation, 5. active protein-losing enteropathy (PLE), and 6. active plastic bronchitis (PB) without remission for ≥6 months [21]. Active PLE was defined by the presence of ongoing diarrhea, and/or edema, and/or pleural effusions, and/or ascites, along with low serum albumin levels (<3.5 g/dL), low total protein (<6.0 g/dL), and increased fecal alpha-1 antitrypsin levels. PB was defined as a hospital admission due to symptoms accompanied by the confirmed presence of fibrinous bronchial casts.

### 2.7. Statistical Analysis

Categorical variables were presented as numbers and percentages, while continuous variables were expressed as medians with interquartile ranges (IQR). A chi-squared test was employed for categorical data analysis. For continuous variables, Student’s *t*-tests were employed for normally distributed data with homogeneous variances (assessed by Levene’s test) and Mann–Whitney U-tests for non-normally distributed data. Competing risk analyses were performed using the Aalen–Johansen estimator to calculate cumulative incidence rates, with death/heart transplantation and interventions for the Fontan pathway as competing risks. Group comparisons were performed using Gray’s test, and hazard ratios were estimated using Fine–Gray competing risk regression models. Survival and freedom from Fontan pathway interventions were calculated using the Kaplan–Meier method, with differences between groups determined via the log-rank test. Risk factors for TCPC pathway interventions were assessed using Cox’s regression models. Statistical significance was set at *p* < 0.05. All statistical analyses were performed using SPSS version 28.0 for Windows (IBM, Ehningen, Germany) and the R-statistical software version 4.3.0 (R Foundation for Statistical Computing, Vienna, Austria).

## 3. Results

### 3.1. Patient Characteristics

A total of 650 patients were included in this study. During the median follow-up of 7.1 (IQR: 2.1–16.1) years, 136 (20.9%) patients presented with TCPC pathway obstruction and underwent surgical/catheter interventions with a median interval of 0.26 (0.03–6.75) years after TCPC. Patient characteristics with and without intervention for TCPC pathway obstruction are detailed in Table 1. Patients who underwent interventions for TCPC pathway obstruction had a higher prevalence of hypoplastic left heart syndrome (40.4 vs. 23.5%, *p* < 0.001), dominant right ventricle (RV, 63.2 vs. 50.2%, *p* = 0.007), and a lower prevalence of univentricular heart (14.0 vs. 22.4%, *p* = 0.031) and tricuspid atresia (10.3 vs. 17.5%, *p* = 0.041) than those who did not. Regarding stage I palliation, the Norwood-type procedure (64.7 vs. 37.2%, *p* < 0.001) and the PDA stent (9.6 vs. 5.1%, *p* = 0.049) were more common in patients who underwent Fontan pathway intervention. The aortopulmonary shunt (20.6 vs. 31.5%, *p* = 0.013) and pulmonary artery banding (8.8 vs. 15.8%, *p* = 0.040) were less common in patients who underwent interventions for TCPC pathway obstruction. The median age at TCPC (2.2 vs. 2.3 years, *p* = 0.003) and BCPS (4.5 vs. 5.5 months, *p* < 0.001) were lower in patients who underwent interventions for TCPC pathway obstruction than in those who did not. Pre-TCPC cardiac catheterization data are shown in Table 2. PAP (median 10 vs. 9 mmHg, *p* < 0.001), LAP (6 vs. 5mmHg, *p* = 0.011), and systemic ventricular pressure (86 vs. 83 mmHg, *p* = 0.012) were higher in patients who underwent TCPC pathway intervention compared to those who did not, whereas, the PA index (150 vs. 174 mm^2^/m^2^, *p* = 0.002), left-PA index (50 vs. 65 mm^2^/m^2^, *p* < 0.001), and the PA symmetry index (0.50 vs. 0.60, *p* = 0.011) were lower in patients who underwent a TCPC pathway intervention compared to those who did not.

### 3.2. Perioperative Data

Table 3 shows perioperative variables and hospital data at TCPC in patients with and without TCPC pathway interventions. There was no significant difference in operative data in patients with and without TCPC pathway interventions except cardiopulmonary bypass time (median 74 vs. 65 min, *p* = 0.008). Regarding postoperative data, the length of intensive care unit stay (7 vs. 6 days, *p* = 0.020) and hospital stay (24 vs. 18 days, *p* < 0.001) were significantly longer in patients with interventions for TCPC pathway obstruction compared to those without. The prevalence of prolonged pleural effusion for more than 7 days (66.2 vs. 44.9%, *p* < 0.001), chylothorax (34.6 vs. 18.2%, *p* < 0.001), and ascites needing drainage (31.6 vs. 15.9%, *p* < 0.001) were higher in the TCPC pathway intervention group compared to the non-intervention group.

### 3.3. Incidence, Location of, and Treatment for TCPC Pathway Obstruction

**Figure 1:**
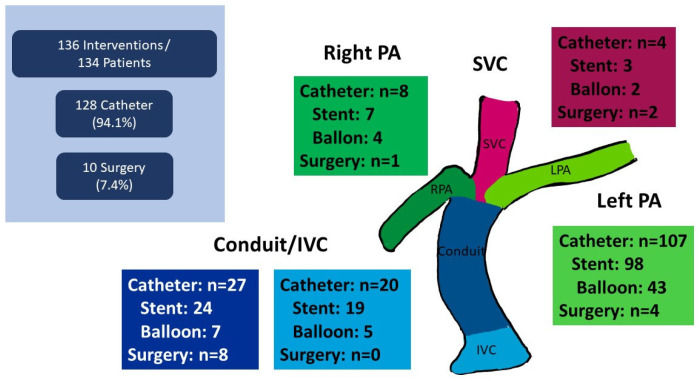
Incidence and location of catheter/surgical interventions for TCPC pathway obstruction. Purple: SVC, dark green: RPA, light green: LPA, dark blue: conduit, light blue: IVC.

Out of the 136 patients who received interventions for TCPC pathway obstruction, 10 (7.4%) patients had surgical interventions with a median interval of 17 (IQR: 8–144) days, and 128 (94.1%) had catheter interventions with a median interval of 0.45 (IQR: 0.03–6.75) years (Table 4 and Figure 1). Both surgical and catheter interventions were needed in two patients. A competing plot of death and interventions for TCPC pathway obstruction is shown in Supplementary Figure S1. Freedom from interventions for Fontan pathway obstruction at 1, 3, 5, and 10 years was 87.7, 85.3, 83.6, and 78.5%, respectively. The surgical procedure included eight conduit revisions, four left-PA reconstructions, two SVC reconstructions, and one right-PA reconstruction. Among the 128 catheter interventions, target lesions included 107 left PAs (Supplementary Figure S2), 9 right PAs, 27 extra-cardiac conduits (Supplementary Figure S3), 20 inferior vena cava (IVCs, Supplementary Figure S4), 8 right PAs, and 4 SVCs. Twelve patients underwent stent implantation both for the left PA and IVC (Supplementary Figure S5). As for the left-PA interventions, balloon dilatation was performed in 43 patients and stent implantation in 98 patients. Era analysis was performed using the early era (1994–2010, n = 320) and the late era (2011–2023, n = 330) (Supplementary Table S2). As a result, the incidence of interventions for TCPC pathway obstruction was higher in the late era than in the early era (24.5% vs. 17.2%, *p* = 0.021). As for the timing of interventions, we performed an additional analysis comparing early intervention vs. late intervention using the cut-off value of 30 days (Supplementary Table S3). The results are shown in Supplementary Tables S1 and S2. As risks for early intervention, the Norwood/DKS procedure (HR: 1.940, *p* = 0.041) and pre-TCPC high EDP (HR: 1.192, *p* = 0.012) were identified as independent factors. As a risk factor for late intervention, pre-TCPC PA intervention was identified as an independent risk factor (HR: 4.380, *p* = 0.009).

### 3.4. Risk Factors for TCPC Pathway Intervention

**Figure 2:**
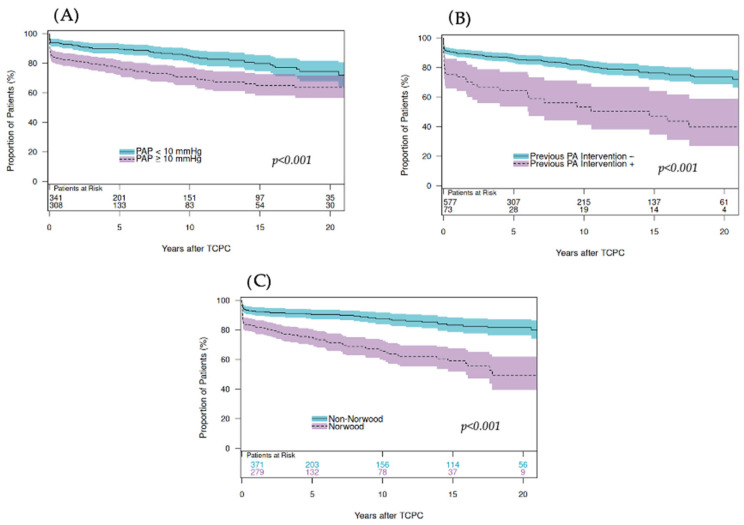
Freedom from TCPC pathway interventions in patients with high PAP (10 mmHg or more) and those without (<10 mmHg) (**A**), with and without previous PA interventions (**B**), with and without previous Norwood procedure (**C**). TCPC: total cavopulmonary connection. PAP: pulmonary artery pressure.

**Figure 3:**
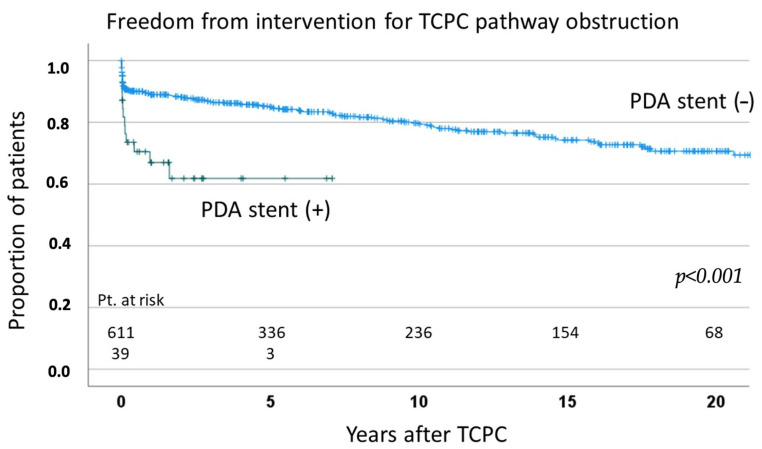
Freedom from TCPC pathway interventions in patients with and without previous PDA stent. TCPC: total cavopulmonary connection, PDA: patent ductus arteriosus.

The results of the Cox regression model are presented in Table 5. The multivariate model revealed previous Norwood procedure (HR: 2.228, *p* = 0.003), previous PDA stent (HR: 2.574, *p* < 0.001), previous PA interventions (HR: 2.514, *p* < 0.001), and higher PAP before TCPC (HR: 1.161, *p* = 0.004) as independent risk factors (Table 5). Freedom from TCPC pathway intervention was significantly lower in patients after the Norwood procedure (*p* < 0.001, Figure 2C), after PDA stent (HR: 2.574, *p* < 0.001, Figure 3), in patients who had previous PA interventions (*p* < 0.001, Figure 2B), and pre-TCPC PAP 10mmHg or more (*p* < 0.001, Figure 2A). When the risk factors for intervention for the left-PA stenosis were analyzed, the multivariate model revealed previous Norwood procedure (HR: 3.476, *p* < 0.001), previous PDA stent (HR: 4.049, *p* < 0.001), previous PA interventions (HR: 2.323, *p* = 0.005), and high PAP before TCPC (HR: 1.158, *p* = 0.010) as independent risk factors (Table 5). When the risk factors for intervention for the conduit/IVC intervention were analyzed, the multivariate model revealed high PAP before TCPC (HR: 1.085, *p* = 0.021) as an independent risk factor (Table 5). As other locations for Fontan pathway intervention are rare and the number of patients undergoing such procedure in this study is limited, no separate analysis on RPA and SVC interventions was conducted. Impact of TCPC pathway interventions on late morbidities in patients who underwent interventions for TCPC pathway obstruction had a higher incidence of PLE (16.0 vs. 2.0%, *p* < 0.001), PB (8.3 vs. 0.8%, *p* < 0.001), and failing Fontan (28.6 vs. 7.6%, *p* < 0.001) compared to those who did not. Cox’s regression analysis revealed that interventions for TCPC pathway obstruction were a significant risk for PLE (HR: 7.181, *p* < 0.001), 264 PB (HR: 8.969, *p* < 0.001), and failing Fontan (HR: 4.294, *p* < 0.001).

## 4. Discussion

### 4.1. Summary of the Results

TCPC pathway obstruction needing intervention was observed in 21% of the patients, and the most common site for pathway obstructions was the left PA. A total of 94% of patients underwent trans-catheter interventions, whereas 7% were treated surgically, most commonly within the first month after TCPC. Previous Norwood procedure, PDA stenting, PA interventions before TCPC, and high PAP at TCPC were identified as risk factors.

### 4.2. Incidence of TCPC Pathway Obstruction

TCPC pathway obstruction is a cause for Fontan failure, found in approximately 12.5% of patients presenting with failing Fontan circulation [10]. McGovern et al. reported that intervention for Fontan pathway obstruction was performed in 13% of patients after TCPC [11]. In this study, 21% of patients underwent intervention for Fontan pathway obstruction. This rate is higher than described in previous reports. We assume that the indication for catheter interventions has become broader as various kinds of new devices have been developed, including re-expandable stents and covered stents, and due to the development of new implantation techniques [22,23,24]. Another reason might be that we treated more complex patients than before. For example, more patients reached TCPC after the Norwood procedure due to advances in operative techniques and pre- and postoperative management, including prenatal diagnosis and interstage home monitoring programs. As our results showed, patients who have undergone the Norwood procedure have a small left PA and often require interventions [25,26,27]. Recently, PDA stenting has emerged as an alternative to the surgical aorto-pulmonary shunts and has become a standard for initial palliation. However, PDA stents have an intrinsic risk for left-PA stenosis because the distal end of the PDA stent is mostly located in the right PA. In this study, a previous PDA stent is identified as a strong risk factor for left-PA intervention after TCPC. Careful management of PA development in patients after initial PDA stenting is mandatory. As for the location of the obstruction, the left PA was the most common lesion, and more than half of the interventions in this study were performed on the left PA. The second most frequent lesion was the extra-cardiac conduit/IVC. Several recent studies have demonstrated a progressive reduction in the cross-sectional area of the extra-cardiac conduit and an associated need for interventions [13,28,29,30].

### 4.3. Surgical vs. Interventional Therapy: Timing and Treatment Options

Early detection of Fontan pathway obstruction and the following intervention are crucial to improve patients’ symptoms and to avoid pressure changes from spiraling into Fontan-associated liver disease and/or heart failure, possibly leading to heart transplantation. In this study, the median period from TCPC to surgical intervention was 17 days, and the period to catheter intervention was 0.5 years. Surgery was chosen mainly for acute obstructions due to thrombus or mechanical distortion following TCPC. A trans-catheter procedure was performed for chronic obstruction. As the literature on reintervention for Fontan pathway obstruction is sparse, presenting a re-intervention rate is difficult. Dowing et al. demonstrated that the overall freedom from surgical and catheter intervention after the Fontan procedure was about 63% and 50% after 20 years [31]. They concluded that two-thirds of patients required surgical or catheter-based re-interventions within 20 years and that the Fontan procedure is typically not the final stage of single-ventricle palliation. Our results, which only evaluated the TCPC pathway obstruction, support this statement. Surgical and interventional treatments can both be favorable in different settings. The threshold for catheter intervention is significantly lower as patients do not have to undergo another sternotomy. Stenting can be a feasible alternative or a bridge to postpone surgery in certain patients, reducing the Fontan gradient and increasing functional capacity [13,29,30,32]. In certain patients, surgical treatment is mandatory due to anatomical reasons or severe stenosis not suitable for interventional stenting.

### 4.4. Left-PA Stenting

Candidates for the Fontan procedure frequently exhibit uneven pulmonary artery (PA) development, with the left PA often being smaller as a result of multiple contributing factors [33,34]. A small left PA can be caused by a narrow aorto-pulmonary space after complex aortic reconstruction (such as DKS or the Norwood procedure) [35]. A PDA stent might be another cause of secondary left-PA stenosis. This study also showed that a previous Norwood procedure and a previous PDA stent were risks for Fontan pathway interventions. Bronchial compression is a well-known complication after the left-PA stent [36]. However, we did not notice this complication in this study cohort. While our study focused on the impact of left-PA stenting, we acknowledge that Fontan failure is multifactorial. Our analysis revealed that patients with left-PA stents had a significantly higher incidence of PLE, PB, and failing Fontan.

### 4.5. Impact of Fontan Pathway Obstruction on Late Outcomes

Few studies have investigated the impact of Fontan pathway obstruction on late outcomes after TCPC. Van Puyvelde et al. showed that Fontan pathway obstruction was the underlying cause in 12.5% of all failing Fontan instances [10]. However, the long-term outcome of Fontan pathway interventions remains to be fully elucidated. In this study, patients with Fontan pathway interventions were associated with an increased incidence of PLE, PB, and failing Fontan, and patients with Fontan pathway interventions had a significantly lower PA index at the time of TCPC. Consequently, our findings indicate that a reduced PA diameter may elevate the risk of late complications—such as PLE, PB, or Fontan failure—through multiple pathophysiological mechanisms. These include increased pulmonary artery and lymphatic pressures resulting from imbalanced pulmonary blood flow, peri-bronchial edema, and exacerbation of lymphatic stasis [37]. Multiple studies have demonstrated improvement in plastic bronchitis (PB) symptoms following interventions targeting the Fontan pathway [38]. Although multiple factors contribute to the development of PLE and PB, and our results do not prove a direct causal relationship between Fontan pathway obstruction and PLE/PB, the evidence supports a clinically significant association between Fontan pathway obstruction and PLE/PB in Fontan patients.

### 4.6. Future Prospective

Interventions for Fontan pathway obstruction after TCPC might increase in the future. Emerging technologies, such as PDA stenting, increase early survival in patients with univentricular hearts. However, the requirement for late interventions might also increase. Along with the development of new devices, indications for interventions might be widened. Maintaining optimal Fontan circulation will likely require more frequent interventions. As high PAP before TCPC was a significant risk factor for both PA and conduit/IVC interventions, this finding emphasized again that maintaining a low PAP is crucial for a successful Fontan outcome. Recent studies have focused on developing stent technologies that reduce the risk of Fontan pathway interventions and accommodate growth in pediatric patients [23,24]. By elucidating these aspects, trans-catheter Fontan pathway interventions are expected to be a more effective and less invasive option. The establishment of tailored follow-up protocols is mandatory. Based on this study, the follow-up protocols after Fontan procedure can be refined for earlier detection of pathway obstruction focusing on signs on high PA pressure and failing Fontan like PLE and PB. We recommend screening patients after Fontan completion closely during the first five years after palliation. High-risk patients who previously received a Norwood procedure, PDA stent, or PA intervention might even benefit from a cardiac catheter examination during the 6-month follow-up.

### 4.7. Limitations

This study has the distinct disadvantages of a retrospective nature and a single-center analysis with inherent bias. Although we examined multiple factors contributing to Fontan pathway obstruction, we were unable to quantitatively assess specific anatomical influences. These include intrinsic factors such as ductal tissue and patent ductus arteriosus (PDA) location; extrinsic compression from procedures like the Damus–Kaye–Stansel (DKS) anastomosis, a dilated ascending aorta, or an enlarged cardiac mass affecting pulmonary venous return; proximal pulmonary artery stenosis secondary to right ventricle-to-pulmonary artery conduits; and pulmonary artery deformation resulting from palliative banding. No adjustments were performed to account for potential confounding variables when comparing follow-up outcomes between patients who underwent pulmonary artery (PA) stenting and those who did not. Consequently, the observed differences between these groups may reflect the influence of underlying factors rather than the effect of stenting alone. The association between pre-Fontan PAP is weak, that the difference in mean pressure between the two groups was small. A more detailed analysis with more patients is required to strengthen this association. Evolving postoperative care or medical management, including anticoagulation protocols and new imaging techniques, may have affected outcomes. The decision on intervention is contingent upon clinical detection, yet not all Fontan pathway obstructions manifest clinically or are referred for diagnostic assessment or therapeutic intervention. This study covers a long period during which new treatment possibilities, such as new types of PA stents and new implantation techniques, have been developed. The complex interaction among these factors and their respective contributions to long-term outcomes necessitates further investigation through prospective, multicenter studies employing standardized imaging protocols.

## 5. Conclusions

In conclusion, interventions for Fontan pathway obstruction were performed in 21% of patients. Left PA stenosis was the primary lesion for TCPC pathway obstruction, most of which were treated with trans-catheter stent implantation. Previous Norwood procedures, PDA stent, PA intervention before TCPC, and high PAP at TCPC were identified as risk factors. As Fontan pathway obstruction was closely associated with late morbidities, special care is needed in these high-risk patients.

## Figures and Tables

**Table 1:** Patient characteristics.

Variables: N(%) or Median (IQR)	Total Cases	TCPC Intervention (−)	TCPC Intervention (+)	*p*-Value
Number of patients	650	514 (79.1)	136 (20.9)	
Age at TCPC (years)	2.3 (1.8–3.3)	2.3 (1.8–3.5)	2.2 (1.8–2.7)	**0.003**
Weight at TCPC (kg)	12.0 (10.7–14.0)	12.0 (10.8–14.4)	11.3 (10.5–13.3)	0.092
Primary diagnosis				
HLHS	176 (27.1)	121 (23.5)	55 (40.4)	**<0.001**
UVH	134 (20.6)	115(22.4)	19 (14.0)	**0.031**
TA	104 (16.0)	90 (17.5)	14 (10.3)	**0.041**
DILV	96 (14.8)	72 (14.0)	24 (17.6)	0.287
PAIVS	34 (5.2)	31 (6.0)	3 (2.2)	0.075
ccTGA	32 (4.9)	27 (5.3)	5 (3.7)	0.450
UAVSD	27 (4.2)	19 (3.7)	8 (5.9)	0.256
Others	48 (7.4)	38 (7.4)	10 (7.4)	0.987
Dominant right ventricle (RV)	344 (52.9)	258 (50.2)	86 (63.2)	**0.007**
Associated cardiac anomaly				
TGA	215 (33.1)	178 (34.6)	37 (27.2)	0.102
DORV	83 (12.8)	70 (13.6)	13 (9.6)	0.207
CoA	83 (12.8)	58 (11.3)	25 (18.4)	**0.027**
Dextrocardia/situs inversus	58 (8.9)	47 (9.1)	11 (8.1)	0.701
Heterotaxy	49 (7.5)	38 (7.34	11 (8.1)	0.785
TAPVC/PAPVC	42 (6.5)	29 (5.6)	13 (9.6)	0.098
Systemic venous return anomaly	61 (9.4)	53 (10.3)	8 (5.9)	0.115
Palliation and pre-Fontan condition				
Norwood/DKS	279 (42.7)	191 (37.2)	88 (64.7)	**<0.001**
AP Shunt	190 (29.2)	162 (31.5)	28 (20.6)	**0.013**
PAB	93 (14.3)	81 (15.8)	12 (8.8)	**0.040**
PDA stent	39 (6.0)	26 (5.1)	13 (9.6)	**0.049**
Prior BCPS	601 (92.5)	474 (92.2)	127 (93.4)	0.647
Age at BCPS (months)	5.1 (3.6–9.6)	5.5 (3.7–11.3)	4.5 (3.3–6.5)	**<0.001**
Weight at BCPS (kg)	5.7 (4.8–7.1)	5.8 (4.9–7.4)	5.3 (4.5–6.2)	**0.009**

TCPC: total cavopulmonary connection; HLHS: hypoplastic left heart syndrome; PAIVS: pulmonary atresia and intact ventricular septum; ccTGA: congenitally corrected TGA; UAVSD: unbalanced atrioventricular septal defect; TGA: transposition of the great arteries; DORV: double outlet right ventricle; CoA: coarctation of the aorta; TAPVC; total anomalous pulmonary venous connection; PAPVC: partial anomalous pulmonary venous connection; PDA: patent ductus arteriosus, BCPS: bidirectional cavopulmonary shunt. Bold indicates statistical significance.

**Table 2:** Cardiac catheterization data before TCPC.

Variables:	Total Cases	TCPC Intervention (−)	TCPC Intervention (+)	*p*-Value
Number of patients	650	514 (79.1)	136 (20.9)	
Hemoglobin (g/dl)	15.4 (14.2–16.7)	15.4 (14.2–16.7)	15.5 (141.-16.9)	0.759
Hemodynamic				
PAP (mmHg)	9 (8–12)	9 (8–11)	10 (8–12)	**<0.001**
LAP (mmHg)	5 (4–7)	5 (4–7)	6 (4–8)	**0.011**
TPG (mmHg)	4 (3–5)	4 (3–5)	4 (3–5)	0.054
SVP (mmHg)	83 (77–93)	83 (76–91)	86 (79–96)	**0.012**
EDP (mmHg)	8 (6–9)	7 (6–9)	8 (6–10)	0.110
MAP (mmHg)	58 (52–64)	58 (51–64)	59 (52–65)	0.454
SO2 (%)	83 (80–86)	83 (80–86)	82 (80–86)	0.237
PA size and balance				
PAI (mm^2^/m^2^)	167 (132–215)	174 (139–222)	150 (114–188)	**0.002**
Right PAI (mm^2^/m^2^)	104 (77–137)	107 (81–138)	94 (73–127)	0.179
Left PAI (mm^2^/m^2^)	60 (43–87)	65 (46–90)	50 (37–64)	**<0.001**
Left-to-right ratio	0.60 (0.39–0.85)	0.61 (0.40–0.90)	0.50 (0.34–0.79)	0.102
Symmetry index	0.58 (0.39–0.77)	0.60 (0.40–0.77)	0.50 (0.34–0.73)	**0.011**
Data are shown by N (%) or median (IQR)				

PAP: pulmonary artery pressure, LAP: left-atrial pressure, TPG: trans-pulmonary gradient, SVP: systolic ventricular pressure, EDP: end-diastolic pressure, MAP: mean arterial pressure, PAI: pulmonary artery index. Bold indicates statistical significance.

**Table 3:** Perioperative variables.

Variables	Total Cases	TCPC Intervention (−)	TCPC Intervention (+)	*p*-Value
Number of patients	650	514 (79.1)	136 (20.9)	
Operative data				
Type of TCPC				
Intracardial	50 (7.7)	43 (8.4)	7 (5.1)	0.210
Extra-cardiac	600 (92.3)	471 (91.6)	129 (94.9)	
Conduit diameter (mm)				
14	1 (0.2)	1 (0.2)	0 (0.0)	0.217
16	9 (1.5)	7 (1.5)	2 (1.6)	
18	518 (86.3)	399 (84.7)	119 (92.2)	
20	57 (9.5)	50 (10.6)	7 (5.4)	
22	15 (2.5)	14 (3.0)	1 (0.8)	
CPB time (minutes)	67 (48–101)	65 (47–99)	74 (56–109)	**0.008**
Aortic cross clamp (AXC)	166 (25.5)	136 (26.5)	30 (22.1)	0.295
AXC time (minutes)	46 (26–73)	47 (26–73)	41 (24–73)	0.901
Fenestration at TCPC	60 (9.2)	45 (8.8)	15 (11.0)	0.415
Concomitant procedure	175 (26.9)	138 (26.8)	37 (27.2)	0.933
DKS	17 (2.6)	12 (2.3)	5 (3.7)	0.383
AVV procedure	79 (12.2)	63 (12.3)	16 (11.8)	0.876
PA reconstruction	60 (9.2)	44 (8.6)	16 (11.8)	0.251
Atrioseptectomy	31 (4.8)	28 (5.4)	3 (2.2)	0.115
SAS/VSD enlargement	14 (2.2)	12 (2.3)	2 (1.5)	0.537
Pacemaker implant	13 (2.0)	10 (1.9)	3 (2.2)	0.847
Postoperative data				
ICU stay (days)	6 (4–9)	6 (4–8)	7 (4–11)	**0.020**
Hospital stay (days)	20 (14–27)	18 (14–26)	24 (18–35)	**<0.001**
Complications				
Pleural effusion	318 (49.4)	228 (44.9)	90 (66.2)	**<0.001**
Chylothorax	139 (21.7)	92 (18.2)	47 (34.6)	**<0.001**
Ascites	124 (19.2)	81 (15.9)	43 (31.6)	**<0.001**
Secondary fenestration	11 (1.7)	7 (1.4)	4 (2.9)	0.204
Follow-up data				
PLE	31 (5.0)	10 (2.0)	21 (21.2)	<0.001
PB	15 (2.4)	4 (0.8)	11 (8.3)	<0.001
Failing Fontan	76 (12.0)	38 (7.6)	38 (28.6)	<0.001

Variables are presented in N (%) or median (IQR). PA: pulmonary artery; TCPC: total cavopulmonary connection; CPB: cardiopulmonary bypass; DKS: Dames–Kaye–Stansel anastomosis; AVV: atrioventricular valve; SAS: subaortic stenosis; VSD: ventricular septal defect; ICU: intensive care unit, PLE: protein-losing enteropathy, PB: plastic bronchitis. Bold indicates statistical significance.

**Table 4:** Interventions for TCPC pathway obstruction.

Variables	N (%)
Number of patients	136
Surgical interventions	10 (7.4)
LPA patch	4 (2.9)
RPA patch	1 (0.7)
Conduit revision	8 (5.9)
SVC patch	2 (1.5)
Catheter interventions	128 (94.1)
LPA intervention	107 (78.7)
LPA balloon	43 (31.6)
LPA stent	98 (72.1)
RPA interventions	8 (5.9)
RPA balloon	4 (2.9)
RPA stent	7 (5.1)
Conduit interventions	27 (19.9)
Conduit balloon	7 (5.1)
Conduit stent	24 (17.6)
IVC interventions	20 (14.7)
IVC balloon	5 (3.7)
IVC stent	19 (14.0)
SVC interventions	4 (2.9)
SVC balloon	2 (1.5)
SVC stent	3 (2.2)
Both surgical and catheter intervention were needed in 2 patients	

TCPC: total cavopulmonary connection, LPA: left pulmonary artery, RPA: right pulmonary artery, SVC: superior vena cava, IVC: inferior vena cava.

**Table 5:** Risk factors.

Risk Factors for Interventions for TCPC Pathway Obstruction						
Variables		Univariate			Multivariate	
	*p*-Value	HR	95% CI	*p*-Value	HR	95% CI
HLHS	**<0.001**	2.238	1.582–3.166			
Dominant RV	**0.001**	1.795	1.263–2.550			
Norwood/DKS	**<0.001**	3.031	2.121–4.332	**0.003**	2.228	1.303–3.811
PDA stent	**<0.001**	3.125	1.736–6.627	**0.009**	2.574	1.262–5.250
Pre-TCPC PA intervention	**<0.001**	2.864	1.926–4.260	**<0.001**	2.514	1.457–4.337
PAP pre TCPC	**<0.001**	1.084	1.035–1.135	**0.004**	1.161	1.049–1.286
LAP pre TCPC	**0.002**	1.105	1.037–1.177			
EDP pre TCPC	**0.005**	1.090	1.026–1.157			
PA index pre-TCPC	**0.031**	0.996	0.992–1.000			
LPA index	**0.006**	0.989	0.981–0.997			
Age at TCPC	**0.005**	0.903	0.840–0.970			
Risk factors for interventions for left-PA stenosis						
Variables		Univariate			Multivariate	
	*p*-value	HR	95% CI	*p*-value	HR	95% CI
HLHS	**<0.001**	2.734	1.874–3.990			
Dominant RV	**<0.001**	2.063	1.383–3.077			
Norwood/DKS	**<0.001**	4.408	2.876–6.757	**<0.001**	3.476	1.852-6.525
PDA stent	**<0.001**	3.934	2.164–7.153	**<0.001**	4.049	1.931-8.489
Pre-TCPC PA intervention	**<0.001**	2.925	1.893–4.518	**0.005**	2.323	1.284-4.204
PAP pre TCPC	**0.041**	1.058	1.002–1.116	**0.010**	1.158	1.035-1.295
LAP pre TCPC	**0.010**	1.098	1.022–1.180			
EDP pre TCPC	**0.035**	1.075	1.005–1.150			
PA index pre-TCPC	**0.002**	0.993	0.989–0.997			
LPA index	**<0.001**	0.984	0.975–0.993			
Age at TCPC	**0.003**	0.858	0.776–0.948			
Risk factors for interventions for IVC/conduit obstruction						
Variables		Univariate			Multivariate	
	*p*-value	HR	95% CI	*p*-value	HR	95% CI
DILV	**0.037**	2.074	1.046–4.110			
Dominant RV	**0.044**	0.527	0.282–0.983			
PAP pre-TCPC	**0.021**	1.086	1.013–1.166	**0.021**	1.085	1.012–1.164
Weight at TCPC	**0.047**	1.025	1.000–1.051			

TCPC: total cavopulmonary connection, HR: hazard ratio, CI: confidence interval, HLHS: hypoplastic left heart syndrome, DKS: DKS: Damus–Kaye–Stansel, PA: pulmonary artery, PAP: pulmonary artery pressure, LAP: left-atrial pressure, EDP: end-diastolic pressure, LPA: left pulmonary artery, IVC: inferior vena cava, DILV: double inlet left ventricle, RV: right ventricle. Bold indicates statistical significance.

## Data Availability

The data that support the findings of this study are available from the corresponding author upon reasonable request.

## Supplementary Materials

The following supporting information can be downloaded at: https://www.mdpi.com/article/10.3390/jcm14207447/s1, Table S1. Reoperation for TCPC pathway obstruction; Table S2. Risk factors for early interventions for TCPC pathway obstruction; Table S3. Risk factors for late interventions for TCPC pathway obstruction; Figure S1. competing plot of death and interventions for TCPC pathway obstruction; Figure S2. Angiogram images demonstrating the effects of stent implantation in the left PA; Figure S3. Angiogram images demonstrating the effects of stent implantation in the extracardiacconduit; Figure S4. Angiogram images demonstrating the effects of stent implantation in the IVC; Figure S5. Angiogram images demonstrating the effects of stent implantation in the left PA and IVC simultaneously.

## Abbreviations

The following abbreviations are used in this manuscript:

BCPS	bidirectional cavopulmonary shunt
DKS	Damus–Kaye–Stansel
HR	hazard ratio
IQR	interquartile ranges
IVC	inferior vena cava
LAP	left-atrial pressure
NYHA	New York Heart Association
PA	pulmonary artery
PAP	pulmonary artery pressure
PB	plastic bronchitis
PDA	patent ductus arteriosus
PLE	protein-losing enteropathy
SVC	superior vena cava
TCPC	total cavopulmonary connection
