# Supplementary material for: Interventions for Fontan Pathway Obstruction in Patients Following Total Cavopulmonary Connection
Source: J Clin Med. 2025 Oct 21;14(20):7447. doi: 10.3390/jcm14207447 (PMC12565572; doi:10.3390/jcm14207447)
Supplement: Supplementary file 1 [file jcm-14-07447-s001.zip › jcm-3877168-supplementary.pdf]

Supplementary Tables

Supplementary Table S1

No	Diagnosis	Age at TCPC (years)	Type of TCPC	Conduit diameter (mm)	Pathway obstruction	Interval	Procedure	Results
1	Situs inversus, SV, CAVV, TGA, PAPVC, bil. SVC, dextrocardia	9.7	LT		IVC stenose, LPA stenose, left SVC stenose	8m	Pathway revision, left SVC patch	alive
2	DILV, TGA, PS	4.5	EC	16	EC-conduit stenosis (5-10mm)	11y	Conduit exchange (18mm), MV closure	alive
3	UAVSD (RV), TGA, PA, PLSVC	4.4	EC	18	EC-conduit multiple stenosis (5-10mm), SVC stenose	42d	Conduit exchange (18mm), SVC Patch	alive
4	DILV, TGA, ASD	5,1	EC	18	LPA stenosis, suppralvalvular AS	19d	LPA Patch, supraaortic AS relief	alive
5	Tricuspid atresia Ib	4.5	EC	20	IVC stenose, LPA stenose	15d	Pathway revision, LPA patch	alive
6	SV, TGA, MA, TAPVC, bil. SVC	16,9	EC	20	RPA stenosis, severe TR	8d	RPA Patch, TV replacement	death
7	HLHS	2.0	EC	18	EC-conduit stenosis with thrombus	11d	Conduit exchange (18mm)	alive
8	HLHS	2.3	EC	18	EC-conduit stenosis with thrombus	8d	Conduit exchange (18mm)	alive
9	HLHS	2.8	EC	18	EC-conduit stenosis with thrombus	1d	Conduit exchange (20mm)	alive
10	Situs inversus, SV, MA, TGA, PA, TAPVC, bil. SVC, ASD, dextrocardia	3.6	EC	18	EC-conduit stenosis, PA stenosis	3m	Pathway revision (EC conduit and PA conduit)	alive

Supplementary table S2

Supple Table S2. Risk factors for early interventions for TCPC pathway obstruction

Variables	Univariate			Multivariate		
	p-value	HR	95% CI	p-value	HR	95% CI
HLHS	**0.009**	1.947	1.179-.216			
Dominant RV	0.210	1.381	0.834-2.287			
Norwood/DKS	**<0.001**	3.183	1.859-5.452	**0.041**	1.940	1.027-3.663
PDA stent	0.062	2.114	0.963-4.642			
Pre-TCPC PA intervent	**0.001**	2.602	1.457-4.647			
PAP pre TCPC	0.074	1.069	0.994-1.151			
LAP pre TCPC	**0.002**	1.162	1.059-1.274	**0.012**	1.192	1.039-1.368
EDP pre TCPC	**0.026**	1.109	1.013-1.216			
PA index pre TCPC	0.246	0.997	0.993-1.002			
LPA index	**0.021**	0.989	0.979-0.998			

Age at TCPC	0.063	0.888	0.783-1.007			

TCPC: total cavopulmonary connection, HR: hazard ratio, CI: confidence interval, HLHS: hypoplastic left heart syndrome, DKS: DKS: Damus-Kaye-Stansel, PA: pulmonary artery, PAP: pulmonary artery pressure, PDA: patent ductus arteriosus LAP: left atrial pressure, EDP: end-diastolic pressure, LPA: left pulmonary artery, RV: right ventricle

Supplementary table S3

Supple Table S3. Risk factors for late interventions for TCPC pathway obstruction

Variables	Univariate			Multivariate		
	p-value	HR	95% CI	p-value	HR	95% CI
HLHS	**<0.001**	2.678	1.584-4.530			
Dominant RV	**0.002**	2.348	1.377-4.003			
Norwood/DKS	**<0.001**	3.155	1.865-5.338			
PDA stent	0.723	1.439	0.193-10.716			
Pre-TCPC PA intervent	**<0.001**	2.834	1.535-5.235	**0.009**	4.380	1.445-13.277
PAP pre TCPC	**0.007**	1.092	1.024-1.164			
LAP pre TCPC	0.417	1.041	0.944-1.148			
EDP pre TCPC	0.449	1.036	0.945-1.135			
PA index pre TCPC	0.066	0.991	0.981-1.001			
LPA index	**0.036**	0.977	0.957-0.998			
Age at TCPC	0.080	0.924	0.846-1.009			

TCPC: total cavopulmonary connection, HR: hazard ratio, CI: confidence interval, HLHS: hypoplastic left heart syndrome, DKS: DKS: Damus-Kaye-Stansel, PA: pulmonary artery, PAP: pulmonary artery pressure, PDA: patent ductus arteriosus LAP: left atrial pressure, EDP: end-diastolic pressure, LPA: left pulmonary artery, RV: right ventricle

Supplementary Figure S1

Competing plot of death and interventions for TCPC pathway obstruction: cumulative incidence of interventions for TCPC pathway obstruction (red) and mortality (blue). The gray curve indicates patients who survived TCPC without experiencing the interventions for TCPC pathway obstruction. TCPC: total cavopulmonary connection.

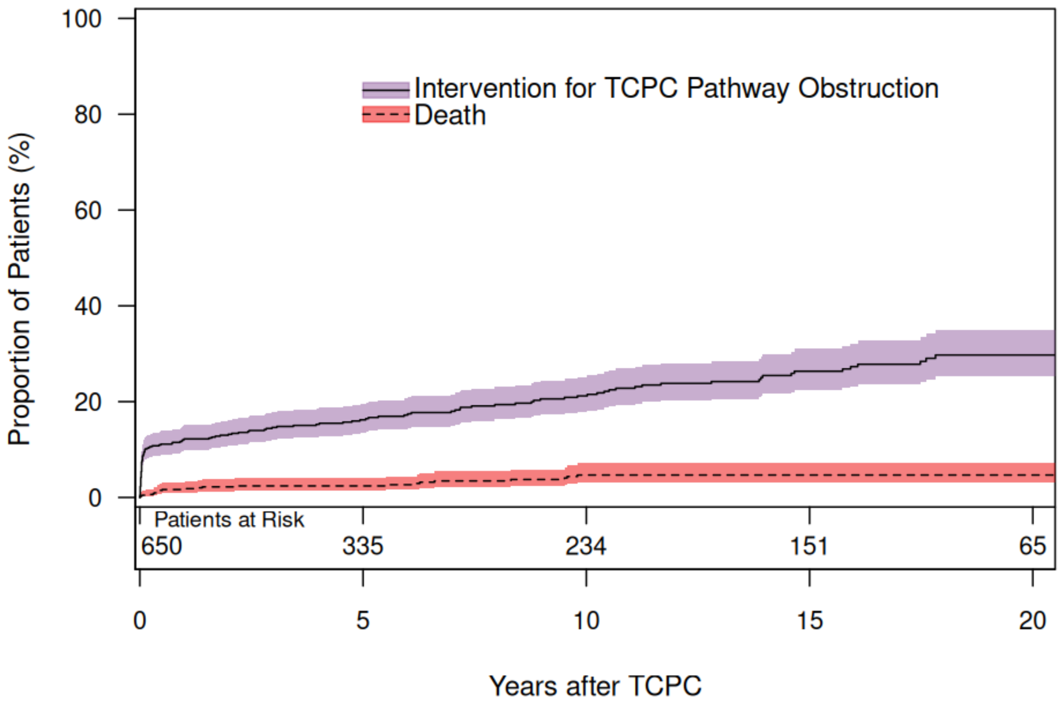

Supplementary Figure S2

Angiogram images demonstrating the effects of stent implantation in the left PA. A small left PA (left) was significantly dilated after the stent implantation (right). PA: pulmonary artery.

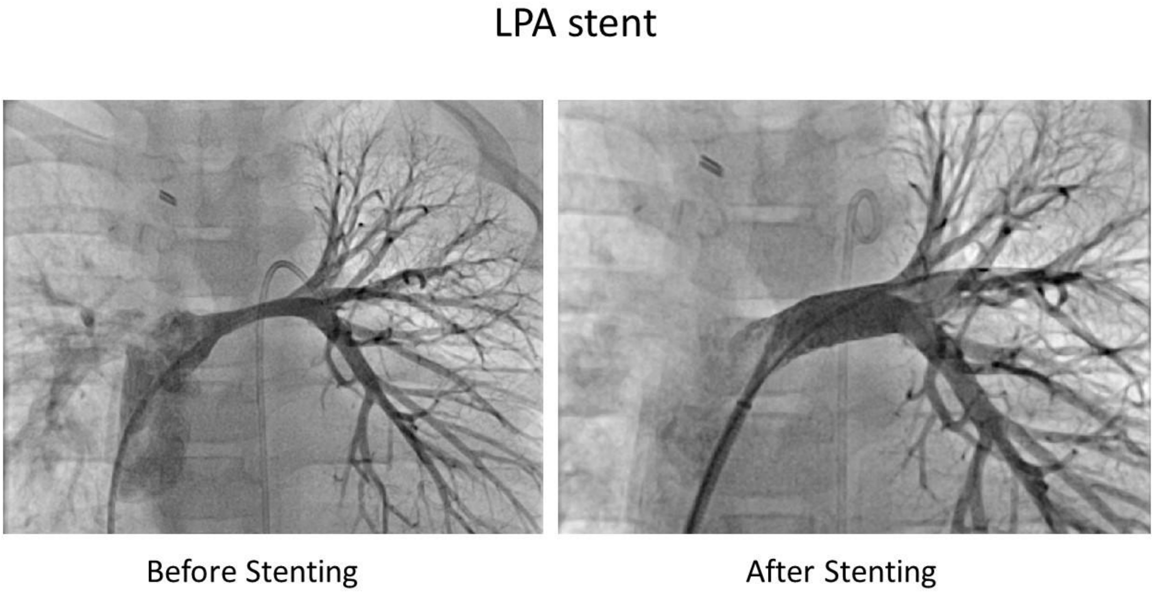

Supplementary Figure S3

Angiogram images demonstrating the effects of stent implantation in the extracardiacconduit. A stenotic extracardiac conduit was significantly dilated after the stent implantation (right).

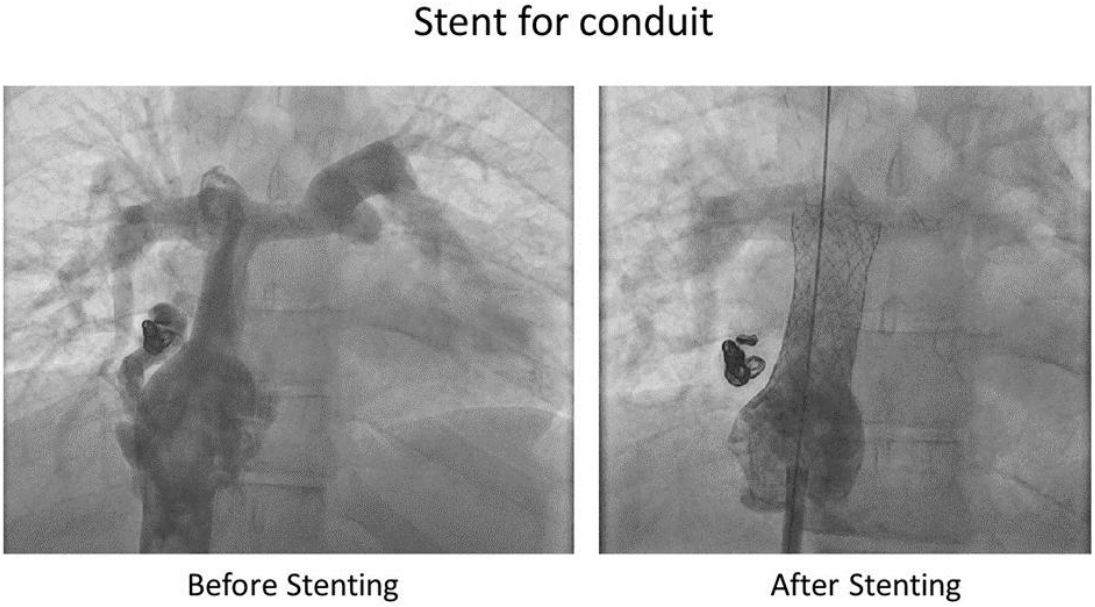

Supplementary Figure S4

Angiogram images demonstrating the effects of stent implantation in the IVC. A stenotic IVC (left) was significantly dilated after the stent implantation (right). IVC: inferior vena cava.

IVC stent

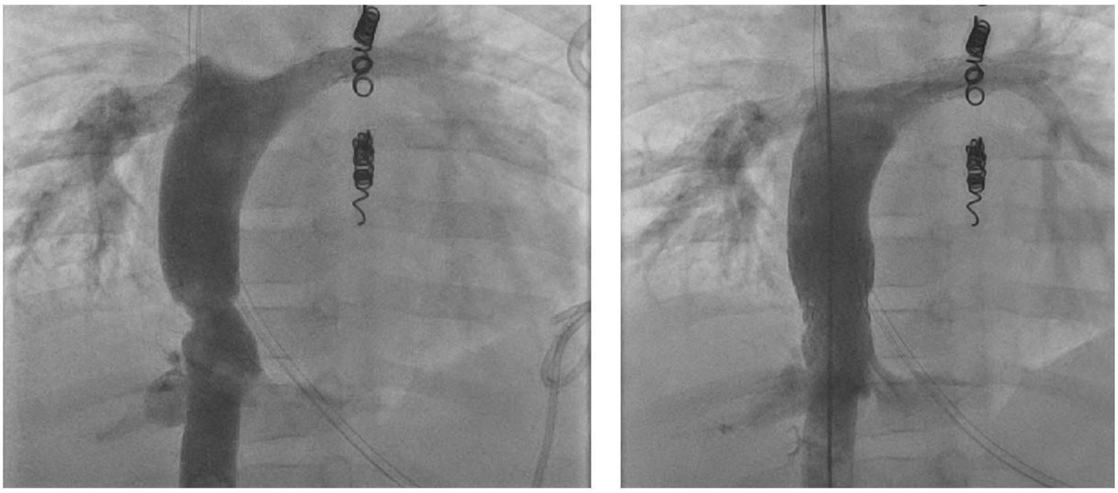

Before Stenting After Stenting

Supplementary Figure S5

Angiogram images demonstrating the effects of stent implantation in the left PA and IVC simultaneously. A stenotic left PA and IVC (left) was significantly dilated after the stent implantation (right). PA: pulmonary artery, IVC: inferior vena cava.

Stent for LPA & conduit

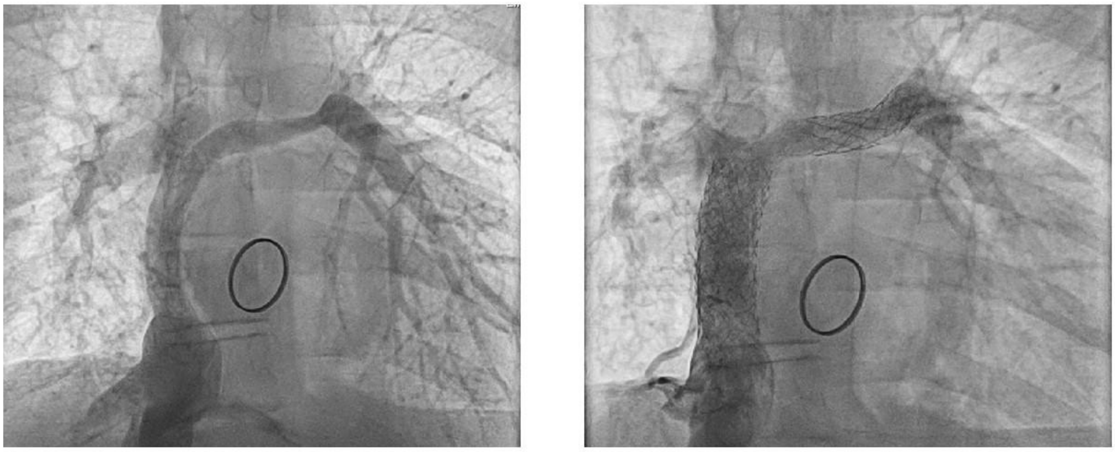

Before Stenting After Stenting
